# PnB Designer: a web application to design prime and base editor guide RNAs for animals and plants

**DOI:** 10.1186/s12859-021-04034-6

**Published:** 2021-03-02

**Authors:** Sebastian M. Siegner, Mehmet E. Karasu, Markus S. Schröder, Zacharias Kontarakis, Jacob E. Corn

**Affiliations:** 1 Department of Biology, ETH Zurich, Zurich, Switzerland; 2 Genome Engineering and Measurement Lab, ETH Zurich, Zurich, Switzerland

**Keywords:** Prime editing, Base editing, Guide RNA design, Web application

## Abstract

**Background:**

The rapid expansion of the CRISPR toolbox through tagging effector domains to either enzymatically inactive Cas9 (dCas9) or Cas9 nickase (nCas9) has led to several promising new gene editing strategies. Recent additions include CRISPR cytosine or adenine base editors (CBEs and ABEs) and the CRISPR prime editors (PEs), in which a deaminase or reverse transcriptase are fused to nCas9, respectively. These tools hold great promise to model and correct disease-causing mutations in animal and plant models. But so far, no widely-available tools exist to automate the design of both BE and PE reagents.

**Results:**

We developed PnB Designer, a web-based application for the design of pegRNAs for PEs and guide RNAs for BEs. PnB Designer makes it easy to design targeting guide RNAs for single or multiple targets on a variant or reference genome from organisms spanning multiple kingdoms. With PnB Designer, we designed pegRNAs to model all known disease causing mutations available in ClinVar. Additionally, PnB Designer can be used to design guide RNAs to install or revert a SNV, scanning the genome with one CBE and seven different ABE PAM variants and returning the best BE to use. PnB Designer is publicly accessible at http://fgcz-shiny.uzh.ch/PnBDesigner/

**Conclusion:**

With PnB Designer we created a user-friendly design tool for CRISPR PE and BE reagents, which should simplify choosing editing strategy and avoiding design complications.

## Background

The landscape of genome engineering has changed drastically since the discovery of the CRISPR (clustered regularly interspaced short palindromic repeats) locus and the associated Cas9 protein [1]. After the successful application of gene editing in human cells, CRISPR-Cas9 research created a totally new and fast-moving field with an exploding number of publications [1, 2]. Directed by a guide RNA (gRNA), the endonuclease activity of Cas9 protein introduces a double strand break (DSB) at the target locus [1]. Each Cas9-induced DSB can be resolved as an error-prone insertion or deletion (indel) or a precise, templated homology directed repair (HDR) event [3]. Fusing various effector domains to catalytically impaired versions of Cas proteins has led to an explosion of tools with outcomes beyond indels and HDR [2].

CRISPR base editors (BEs) use a cytidine- or adenine-deaminase protein to generate a DNA base transitions from C → T or A → G, respectively [4, 5]. While the gRNA bound to the Cas enzyme directs the BE to a genomic locus, the deaminase can edit its target base within a certain window [4, 5]. Base editors have been used in many animal and plant models and hold a great promise for the correction of disease-causing single nucleotide variants (SNVs) in a broad spectrum of diseases [6–11]. Adenine-deaminase base editors (ABEs) in particular exhibit a promising combination of a high efficiency at the on-target site with low off-target DNA and RNA editing [12, 13]. In contrast, some of the early CBEs versions suffered from off-target activity [12], and only the newly engineered versions of these CBEs or Target-AID based CBEs exhibit sufficient specificity to be considered for therapeutic applications [13–15].

Even more recently, Prime Editors (PEs) have introduced the possibility of small programmable genomic changes without HDR. The PE2 consists of a Cas9 nickase fused to an engineered reverse transcriptase [16]. To introduce a modification in the genome, PEs use a prime editing extended guide RNA (pegRNA), consisting of a 20 nt guide sequence, a primer binding site (PBS) and a reverse transcriptase template (RTT) [16]. The guide directs the Cas enzyme to a target site, the PBS hybridizes to the opposite strand to prime the reverse transcriptase, and the RTT integrates the desired genomic alteration. To further optimize PE2, the PE3 system uses an additional guide that directs a nick to the non-edited complementary strand, shifting the equilibrium of DNA repair to favor the edited strand [16]. This secondary nicking can lead to indels if the edited strand is nicked before the RTT sequence has been incorporated, which was recently shown to be the case [17]. However, the PE3b system solves this problem by using a second guide against the anticipated edit, such that the nick on the wild-type strand only occurs after successful sequence incorporation on the edited strand [16]. Therefore, for users who would like to avoid potential off targets, we recommend using either the PE2 or PE3b system.

The manual design of a complex pegRNA and multiple nicking sites can be laborious, challenging and error-prone, especially in these early days of prime editing when optimization may require the design and testing of several pegRNAs. Software to easily design pegRNAs would make this exciting new technology more accessible to a wide user base. Several web-based tools exist for the design of BEs [18–20]. However, to the best of our knowledge there is no publicly available software that can design both base editing and prime editing gRNAs.

Here, we present the PnB Designer tool, a web-based application for gRNA design for PE, CBE, and the most recent ABEs such as ABEmax and ABE8e [21, 22]. PnB Designer provides the user with an intuitive interface to make editing strategy and target site selection straightforward. It generates helpful output commands to lead the user through design problems and presents the resulting guides in a simple and easy to understand fashion. We have also used PnB Designer to design candidate pegRNAs to model all human mutations in ClinVar. Multiple types of ClinVar-targeting pegRNA designs are included to assist in the design of experiments using PEs. PnB Designer compares favorably to other BE design webservers and is the first BE and PE design webserver of which we are aware (Table 1).

**Table 1:** Comparison table between PnB Designer and other guide RNA design applications

Tool	PnB Designer	SNP-CRISPR	BE-FF	beditor	BE-Designer	Benchling
pegRNA design for prime editing	✓	×	×	×	×	×
Protospacer design for prime editing	✓	✓	×	×	×	×
Integrated PAM variants for ABE	7	0	6	6	6	0
Assessment of off-targets	×	✓	✓	✓	✓	✓
Support multiple sample file import	✓	✓	×	✓	✓	×
Explanatory feedback after wrong input	✓	✓	×	✓	×	✓
Guide design on non-reference genome	✓	✓	✓	×	×	×
Access	Webserver	Webserver	Webserver	Installation in python environment and GUI or command line	Webserver	Integrated feature on the benchling website
Input format	Genomic coordinates, text format sequence input by user	Genomic coordinates file only	SNP ID, genomic coordinates, text format sequence input by user	Genomic coordinates file only	Genomic coordinates file, text format sequence input by the user	DNA sequence on the benchling website
URL	fgcz-shiny.uzh.ch/PnBDesigner/	flyrnai.org/tools/snp_crispr	danioffenlab.com/be-ff	pypi.org/project/beditor	rgenome.net/be-designer/	benchling.com
Reference	–	Chen et al. [38]	Rabinowitz et al. [18]	Dandage et al. [19]	Hwang et al. [20]	–

## Implementation

PnB Designer is a web application to design gRNAs for CRISPR prime and base editors. Written in R, PnB Designer is constructed with the Shiny package [23] and Bioconductor [24, 25]. The application includes feedback after each input to help users avoid flaws in the complex design of base and prime editing gRNAs. The user-friendly interface combined with instructions and explanatory pop-up windows in the result visualization make it accessible to users on all levels.

**Fig. 1:**
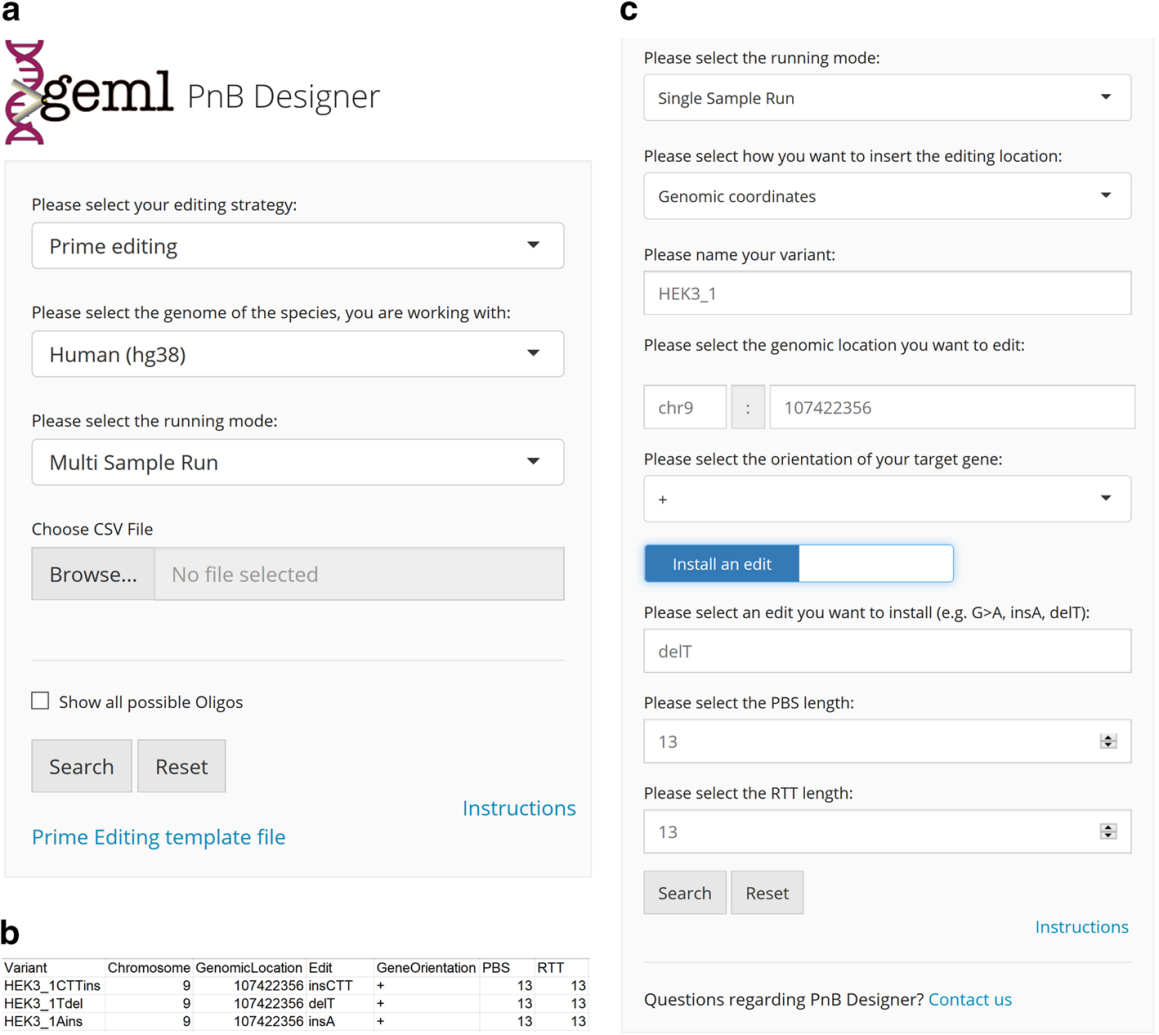
Overview of the User Interface for PnB Designer. **a** Graphical layout of the user interface of the PnB Designer application in the ‘Multi Sample Run’ mode. In the first input fields, the user can select the editing strategy, either base- or prime editing and the running mode. When the ‘Multi Sample Run’ mode is selected, a CSV file with the user specified samples can be loaded into the application for batch analysis. **b** Template CSV file can be downloaded under the ‘Prime Editing template file’ link and has the format shown in panel b. **c** User interface in the ‘Single Sample Run’ mode. The editing location can be defined using genomic coordinates or as a raw text sequence

In each gRNA design run, the user can choose an editing strategy and target genome (Fig. 1a). In the ‘Genomes panel’ users can select genomes of commonly used organisms such as *Homo sapiens, Mus musculus* and *Danio rerio*. Moreover, since genome manipulation applications such as prime and base editing have been successfully deployed in plants [9, 26, 27], *Oryza sativa* (Asian rice), *Arabidopsis thaliana* (Thale cress) and *Vitis vinifera* (Common Grape Vine) are also included in the ‘Genomes panel’. Users can also input their own target sequence by selecting ‘None of the above’ in the ‘Genomes panel’ and ‘Sequence input’ in the later panel, enabling design of gRNAs for non-model organisms or synthetic constructs.

Finally, the user can decide either to design BE or PE gRNAs for a single edit, or to automatically design multiple gRNAs using a comma-separated value (CSV) format file as input (Fig. 1b). After processing the user’s inquiries, PnB Designer allows the user to save the results of a gRNA search by clicking one of the download buttons, located below the output table.

## Results

### Prime editing with PnB Designer user interface

Selecting the ‘Prime editing’ option, the user can install a desired edit or correct a certain mutation of any type (substitution, insertion, deletion) by setting the switch button to the left or right position (Fig. 1c). The user can use genomic coordinates or input a target sequence in text format (Fig. 1c). PBS and RTT length are important parameters for successful pegRNA design. While it is suggested to start with a PBS length of ~ 13 nt and RTT length of 10–16 nt, precise rules for their values have not yet been determined and pegRNA efficiency can be optimized by varying RTT length [16]. Hence, in PnB Designer, PBS and RTT lengths are by default set at suggested values of 13 nt but can be easily modified by the user.

### Design strategy for pegRNAs

PnB Designer scans the sense and antisense strands to find all possible 5′-NGG-3′ protospacer adjacent motif (PAM) sites around the edit position, beginning + 6 nt to the 3′ end of the desired edit and then scanning 100 nt in the 5′ direction, giving the user the option to choose also very distant PAMs. Non-NGG PAMs are currently not explored due to the lack of experimental validation with PEs, but expansion to other PAMs is anticipated and accordingly their support will be added to future versions of PnB Designer. All possible NGG PAMs are stored and evaluated in respect to their distance from the edit position and the input RTT length. A pegRNA is considered a possible candidate if the edit is fully covered by the RTT. PnB Designer then stores the protospacer, PBS, and RTT sequences. The intended edit on the coding strand is also highlighted for confirmation. Nicking guides for the PE3 and PE3b systems are designed and filtered to provide a suitable selection of gRNAs, according to recommendations by the Liu lab [16]. For PE3, only nicking guides 40–100 nt up/downstream of the initial nick are considered. For PE3b, only PAM sequences on the complementary strand that partially overlap with the PE2 PAM or protospacer sequence are displayed, as indicated in the PE3b manual design scheme suggested by the Liu lab.

### Prime editing with PnB Designer: result visualization

**Fig. 2:**
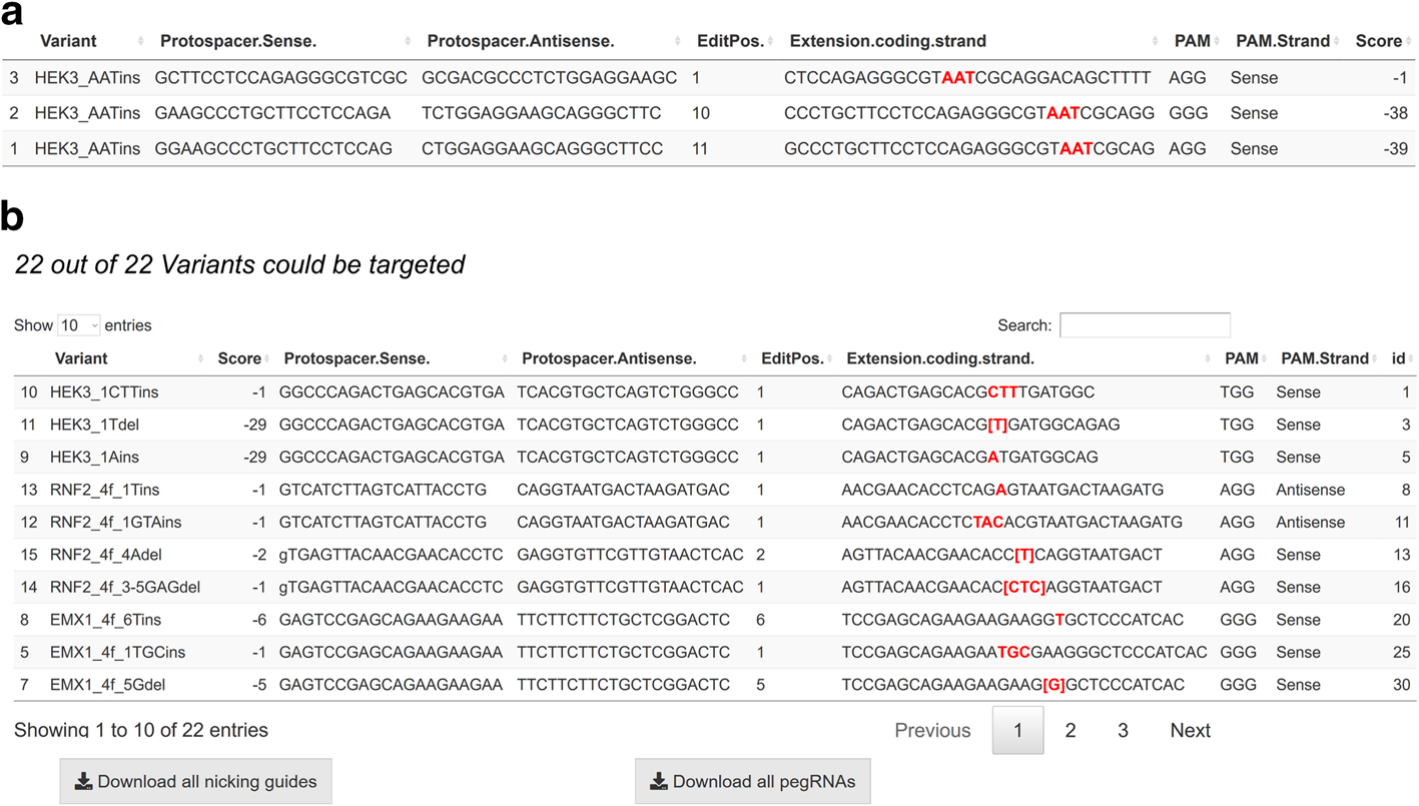
Overview of the pegRNA output tables in the ‘Single Sample Run’ and ‘Multi Sample Run’. **a** ‘Single Sample Run’ output table with all the possible pegRNAs for the selected variant. The edit is visualized with red bold text. Further information on the edit position as well as the computation of the ‘pegRNA-Score’ can be obtained by clicking on each column. **b** ‘Multi Sample Run’ output with overview of variants and output table. The title shows how many variants could be targeted with a possible pegRNA. By default, the pegRNA with the highest ‘pegRNA-Score’ for each variant is displayed. A Download button is displayed over which you can directly download the pegRNA oligos with cloning sites for subsequent ordering

After a successful run, the resulting sequences are shown in an output table (Fig. 2a, b) with the following parameters: the variant name, ‘pegRNA Score’, protospacer sequence, edit position relative to the PAM, PAM sequence, PAM strand and coding strand 3′ extension with implemented edits. As an indicator for the quality of each pegRNA, we implemented a ‘pegRNA Score’ equation based on the recommendations from the Liu lab [16]. Our intent was to automate several heuristic constraints that would otherwise need to be manually evaluated for each pegRNA, allowing the user to triage pegRNAs that are highly unlikely to work. The ‘pegRNA Score’ is thus similar to early scoring systems for normal gRNAs. As with DSB-forming Cas9 editing, large amounts of data on the efficiency of prime editing with many different pegRNAs may in future enable approaches such as machine learning to predict pegRNA efficiency.

The simple ‘pegRNA Score’ was implemented to make users aware of any design features of their pegRNA, which could impede the function of the pegRNA, when used under the conditions described in Anzalone et al. [16]. The pegRNA Score follows the same penalty system as suggested by the Liu lab, with larger negative numbers are indicating worse pegRNA designs. In case of a C as the first base in the 3′ extension editing shows lower efficiency potentially due to disruption of gRNA structure by pairing with G81 of Cas9 [16] and a penalty score of − 28 is given to this pegRNA, in this case increasing the RTT length is recommended. Multiple thymine (T) nucleotides (more than 4) in the 3′ extension of the pegRNA are strongly penalized (score − 50), since the current pegRNA plasmid utilizes an RNA Polymerase III promoter and thymine stretches are recognized as transcription termination signals [28]. The influence of T stretches in a pegRNA was also found as one of the most important features, predicting a decrease in PE2 efficiency in one of the first prime editing screens [29]. Another important point to consider is the number of homologous bases after the intended edit. This should be more than 5 nt, or even more than 10 nt if possible, to increase annealing at that end. Thus, a penalty score of − 6 is assigned if the number of homologous nucleotides is less than five. Lastly, PE is most efficient if the edit is close to the initial nick site. Hence, making pegRNAs with an edit position within the protospacer or PAM is preferable (edit position 1–6) [16], and a penalty of − 1 per increase of the edit position is given to the pegRNA to show deviation from this optimal design. The sum of all penalties defines the ‘pegRNA Score’. Examples of pegRNA designs with favorable (Additional file 1: Figure 1a, first row) and poor scores (Additional file 1: Figure 1a, last two rows) can be seen in the Supplementary information. PegRNAs that receive multiple penalties are highly unlikely to be active, and thus can be omitted from the testing. However, at this early stage of prime editing, we do not recommend using the pegRNA Score as a discriminator between similarly scoring pegRNAs. It is instead advisable for users to adopt loose thresholds and experimentally test multiple pegRNAs spanning a wide range of good scores. For example, all pegRNAs from Anzalone et al. Figure 4a have good pegRNA scores between − 14 and − 1, but there is only a slight correlation between these reasonably-scoring pegRNAs and their efficiency. None of the pegRNAs with good editing efficiency reported by Anzalone et al. have a high pegRNA penalty, supporting the triage of pegRNAs with bad scores (Additional file 2: Figure 2).

From the output table, the user can select a desired pegRNA and, if they wish, additionally implement the PE3 or PE3b system. If so, they will be presented with potential nicking guides according to the design suggestions summarized above. After pegRNA and the optional nicking guide selection, their sequences can be directly downloaded including adapters for cloning into the BsmBI-digested acceptor vector from Anzalone et al. and the pegRNA acceptor vector deposited by the Liu lab on Addgene [#132777].

In the output table of the multi sample mode only the pegRNA with the highest Score for a variant is shown. The user can also access all pegRNAs by selecting the ‘Show all possible pegRNAs’ box (Fig. 1a). For non-targetable variants with the specific input conditions, a row with possible reasons for the unsuccessful search is displayed. Additionally, users can download the oligonucleotides for all pegRNAs with cloning extensions for direct Golden Gate assembly into the pU6-pegRNA-GG-acceptor plasmid [16].

### Automated design of pegRNAs to reconstitute ClinVar genotypes using prime editing

**Fig. 3:**
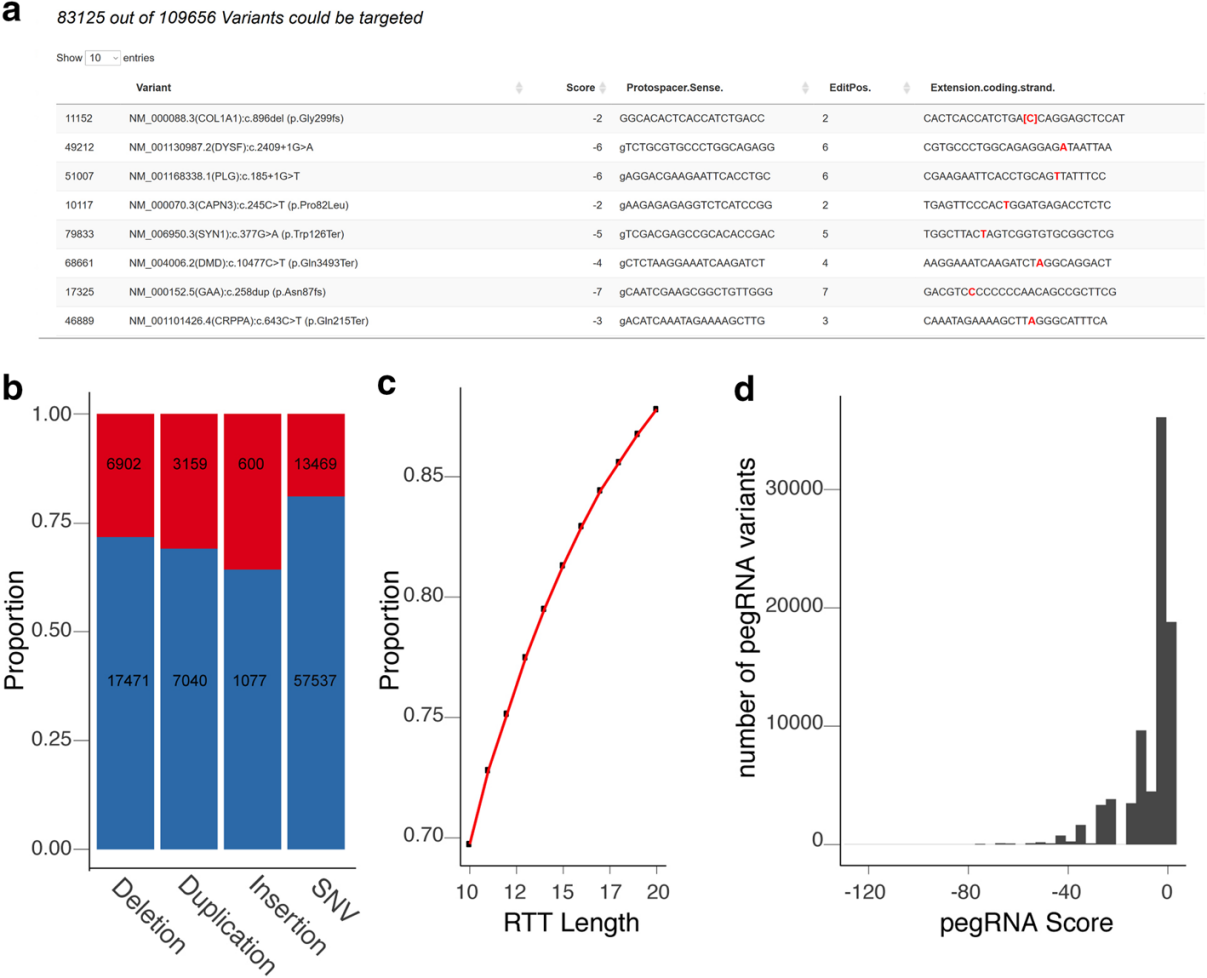
pegRNA design for modelling all pathogenic and potentially pathogenic SNVs, insertions, duplications, and deletions in ClinVar. **a** Section of the output table after designing pegRNAs for a dataset of 109,565 variants. 83,125 variants were targetable using prime editing with a 13 nt RTT. **b** Quantification of different type of variants in the dataset and proportion of variants targetable with PE for each type highlighted in blue, not targetable in red, respectively. The black number in the bars represent the number of variants (RTT length: 13 nt). **c** Proportion of pathogenic and likely pathogenic variants targetable using different lengths of RT templates (10–20 nt). **d** Distribution of the ‘pegRNA-Score’ of the highest possible score for each variant (RTT length: 13 nt). Most pegRNAs have a score greater than − 20, showing that these are possibly functional pegRNAs with no major design penalties. Figures were produced using the package ggplot2 [37] with R (Version 3.6.3)

We used PnB Designer to design candidate pegRNAs to model all known human pathogenic or likely pathogenic nuclear-encoded variants in ClinVar [30] (https://ftp.ncbi.nlm.nih.gov/pub/clinvar/tab_delimited/variant_summary.txt.gz), filtered to target deletions, duplications, insertion and SNVs accessible to PE [16]. We started with an input ClinVar dataset containing 71,006 SNVs, 24,373 deletions, 10,199 duplications and 1677 insertions. We found that with a PBS and RTT length of 13 nt we could target 57,537 out of 71,006 SNVs (81%), 17,471 of 24,373 (72%) deletions, 7040 out of 10,199 (69%) duplications and 1077 out of 1677 (64%) insertions (Fig. 3a, b). Overall, we retrieved suitable pegRNA designs for 78% of the analyzed variants. Since the constraints on RTT length are not well defined experimentally, we repeated this analysis with RTT lengths ranging from 10 to 20 nt. As expected, fewer variants were targetable with a shorter RT template, and at the longest RTT length of 20 nt, we were able to design pegRNAs for 87% of all ClinVar input variants (Fig. 3c). With a RTT length of 13 nt the ‘pegRNA Score’ distribution shows that the majority of pegRNAs have a score of − 20 and higher, which shows that only some pegRNAs suffer from additional penalties (3′ C or multiple thymine in the extension) (Fig. 3d). The frequency of targetable variants did not yet plateau with a 20 nt RTT, but longer RTTs are likely to introduce problems in the already long pegRNA. In summary, we were able to successfully design gRNAs for prime editing for a large dataset of human variants using PnB Designer. All ClinVar-targeting pegRNAs of all RTT lengths can be accessed (Additional files).

### Base editing with PnB Designer: user interface

**Fig. 4:**
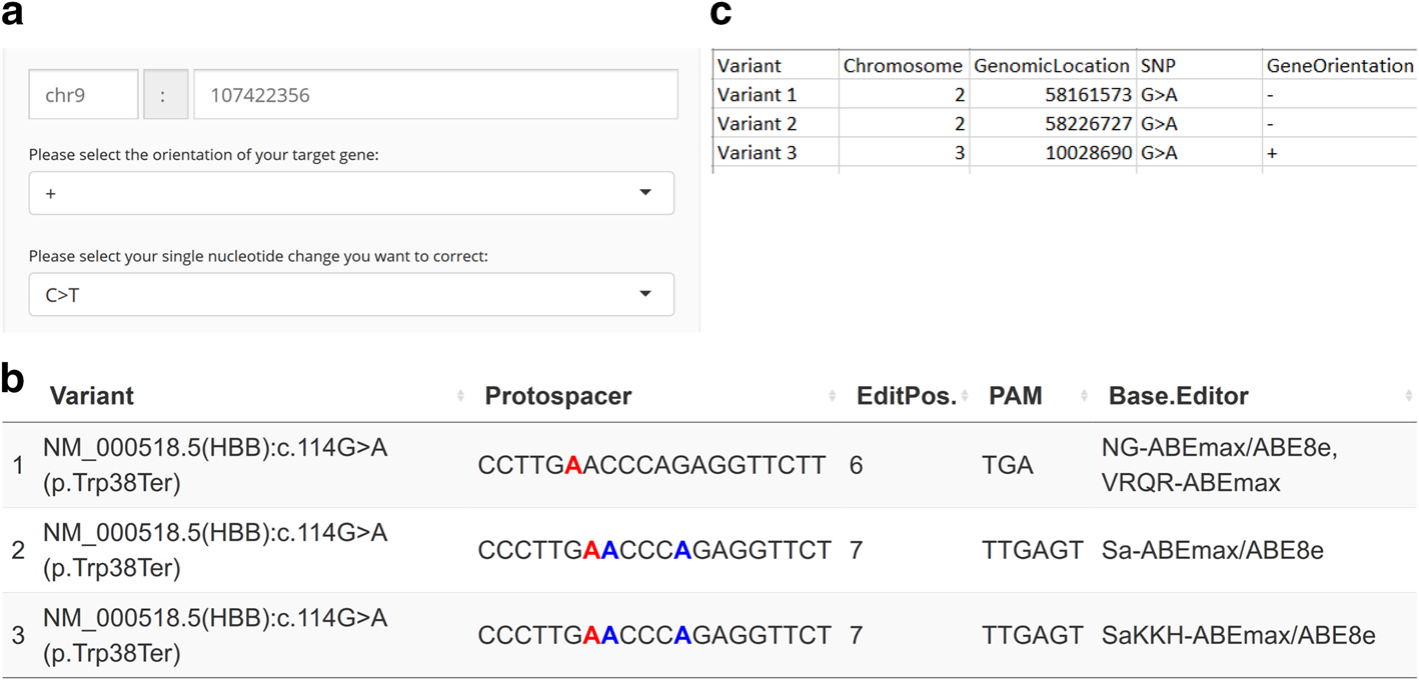
Overview of base editing input and output. **a** Segment of the BE specific user interface. A target sequence in text format can be provided by the user to install an edit. For correcting a specific SNV, genomic coordinates and the orientation of the gene must be provided by the user. C → T or G → A SNVs can be selected for correction. **b** Example ‘Single sample run’ for a random ClinVar variant. The target adenine is highlighted in red. Other adenines in the editing window that could be modified are in blue. Edit position relative to the PAM, PAM sequence and the corresponding ABE are shown. **c** Example base editing template for the ‘Multi sample run’

When selecting the ‘Base editing’ option, the user can either input a target sequence in text format or select genomic coordinates, orientation of the target region and the SNV they want to revert to the reference wildtype sequence (Fig. 4a). The PnB Designer supports gRNA design for ABEs and CBEs. Due to the known off-target propensities of early CBEs, only engineered versions with decreased off-targets have been implemented: BE3 (R33A), BE3 (R33A/K34A), BE3-hA3A (R128A) and Target-AID, all recognizing 5′-NGG-3′ PAMs [13–15]. However, PnB Designer can design base editing gRNAs for ABEs based on SpCas9 (*Streptococcus pyogenes*) and SaCas9 (*Streptococcus aureus*) with a broad spectrum of PAM variants: 5′-NGG-3′ (SpCas9), 5′-NGA-3′ (SpCas9-VRQR), 5′-NGCG-3′ (SpCas9-NG), 5′-NNGRRT-3′ (SaCas9), 5′-NNNRRT-3′ (SaCas9-KKH) and the newly by structure-guided engineering created versions SpG 5′-NGN-3′ and SpRY 5′-NRN-3′ and to a lesser extent 5′-NYN-3′ [5, 31–35].

### Design strategy for BE gRNAs

The user can either enter a sequence or genomic coordinates. During manual sequence input, the user can select the desired single base pair change (for example A>G to convert an adenine at that position into a guanine). With the input of genomic coordinates, PnB Designer retrieves the genomic sequence from the selected reference genome and converts the specific variant sequence to include the SNV. The resulting sequence is searched for PAM sites in the right distance to the SNV given the described editing windows of the Cas9-ABE and -CBE variants [5, 13–15, 31–35]. With ABEs, C → T genomic variants can be reverted by A → G conversion on the antisense strand to achieve the intended edit on the sense strand. Similarly, with CBEs, C → T and G → A conversions are possible. All previously described PAM variants with their respective editing window are tested against the edit, allowing the user to choose the best tool given the desired outcome. The editing windows are defined based on the experimental data. For example ABEmax was implemented with an editing window from base 5–7, with base 1 being the most distal from the PAM site [22]. Additionally for BE3 (R33A/K34A), the strong sequence preference for a 5′ T next to the edit has been included as well [13].

### Base editing with PnB Designer: result visualization

Resulting gRNAs are displayed in the output table, with the target base highlighted in red. Bystander adenines or cytosines in a given editing window that could also be modified are highlighted in blue, giving an indication about potential off-targets of the deaminase at the on-target site. With this information users can easily select the “cleanest” gRNAs (Fig. 4b). The output table consists of the 20 nt protospacer sequence (targeting the variant sequence), edit position, PAM site and suggested base editor that can target this variant. Additional information about the edit position can be found by clicking on the respective column. The newly developed near-PAMless SpCas9 variant SpRY base editor has lower overall efficiency when compared to the other implemented base editors [31]. We included this information in the output table to guide the user to the most efficient editing strategy when multiple ABEs are able to target a site. An example for a good base editing guide with no bystander bases can be seen in the Supplementary Information (Additional file 1: Figure 1b, third row).

## Conclusion

In summary, PnB Designer is a flexible and user-friendly web application for single and batch design (Figs. 1b, 4c) of PE pegRNAs and BE gRNAs. PnB Designer allows users to design pegRNAs with ease, lowering the barrier to entry for PE applications. Additionally, we implemented most recent CBEs and ABEs with a wide variety of PAM variants, which are not yet available in other base editing design webservers (Table 1). While other BE design tools provide custom base editor options to choose new PAMs and editing windows, we think implementation of the newest base editors with their experimentally established editing windows makes it more user friendly. As the base editor field is rapidly moving forward, support for new base editors will be added to the application, as they are developed. For the assessment of off-targets generated by the mismatch tolerance of the sgRNA we recommend using Cas-OFFinder (http://www.rgenome.net/cas-offinder/) [36]. The guide sequences from the output table can be easily transferred and validated, which is especially important for users working on off-target sensitive applications.

Since the ‘pegRNA Score’ represents a first attempt to rank pegRNA activity and is based on early PE applications [16], it should be only used as an indicator. This score will be refined as dependencies on efficient pegRNA design emerge. Moreover, users are advised to test the efficiency of multiple pegRNAs in their experimental models. This is made easy by PnB Designer. Our dataset of pre-designed PE guides for the majority of ClinVar variants using a wide variety of RTT lengths (Additional files), provides an easy reference for experimenters seeking to model a potentially pathogenic human mutation. Increasing the RTT length even further could increase the number of targetable variants, but this leads to a trade-off of extremely long pegRNAs. Finally, we can say that PnB Designer is an innovative web application for gene editing research and could support the rapid development of gene editing strategies for translational research.

## Availability and requirement

- **Project name**: PnB Designer
- **Project home page**: http://fgcz-shiny.uzh.ch/PnBDesigner/
- **GitHub repository**: https://github.com/SebastianSiegner/PnB-Designer
- **Programming language**: R
- **Other requirements**: No requirements
- **License**: No license needed
- **Any restrictions to use by non-academics**: No access restrictions

## Supplementary Information

**Additional file 1.** **Fig. 1.** Example output tables for good and suboptimal guide design for PE and BE. **a** Example output table for prime editing, showing pegRNA design with a high ‘pegRNA Score’ and no additional penalties (row 1), as well as pegRNAs suffering from design flaws and consequently assigned with a low ‘pegRNA Score’ (row 5 and 6). **b** Example output table for base editing. Three different CBEs can target this SNV. However, two of those show bystander bases in the editing window (marked in blue), which could be edited as well (row 1 and 2). Therefore, the use of Target-AID (row 3) should be prioritized for ‘clean’ editing at this locus.

**Additional file 2.** **Fig. 2.** Scatterplot, showing ‘pegRNA Score’ vs. editing efficiency of reported pegRNAs. Reported pegRNAs from Anzalone et al. Fig. 4a with their respective average editing efficiency plotted against their pegRNA Score as calculated by PnB Designer. A linear regression was fitted to investigate the relationship between these two variables and a R^2^ value of 0.297 was obtained. Figure was made using MS Excel (2016).

## Data Availability

The dataset(s) supporting the conclusions of this article is(are) included within the article (and its additional file(s)).

## Abbreviations

ABEs: CRISPR adenine base editors
BEs: CRISPR base editors
Cas9: CRISPR associated 9
CBEs: Cytidine deaminase base editors
CRISPR: Clustered regularly interspaced short palindromic repeats
dCas9: Enzymatically inactive Cas9
DSB: DNA double strand break
gRNA: Guide RNA
HDR: Homology directed repair
indel: Insertion or deletion
nCas9: Cas9 nickase
PAM: Protospacer adjacent motif
PBS: Primer binding site
pegRNA: Prime editing extended guide RNA
PEs: CRISPR prime editors
RTT: Reverse transcriptase template
SNVs: Single nucleotide variants
