# Supplementary material for: PnB Designer: a web application to design prime and base editor guide RNAs for animals and plants
Source: BMC Bioinformatics. 2021 Mar 2;22:101. doi: 10.1186/s12859-021-04034-6 (PMC7923538; doi:10.1186/s12859-021-04034-6)
Supplement: Supplementary file 1 — Additional file 1. Fig. 1. Example output tables for good and suboptimal guide design for PE and BE. a Example output table for prime editing, showing pegRNA design with a high ‘pegRNA Score’ and no additional penalties (row 1), as well as pegRNAs suffering from design flaws and consequently assigned with a low ‘pegRNA Score’ (row 5 and 6). b Example output table for base editing. Three different CBEs can target this SNV. However, two of those show bystander bases in the editing window (marked in blue), which could be edited as well (row 1 and 2). Therefore, the use of Target-AID (row 3) should be prioritized for ‘clean’ editing at this locus. [file 12859_2021_4034_MOESM1_ESM.pdf]

Sup. Fig. 1

A

pegRNA Table

Variant	Score	Protospacer.Sense.	Protospacer.Antisense.	EditPos.	Extension.coding.strand.	PAM	PAM.Strand
ExampleLocus_delT	-1	GGCCCAGACTGAGCACGTGA	TCACGTGCTCAGTCTGGGCC	1	CAGACTGAGCACG[T]GATGGCAGAGGAAAGGAAGCCCTGCTTCCTCCA	TGG	Sense
ExampleLocus_delT	-22	gCTGGCCTGGGTCAATCCTTG	CAAGGATTGACCCAGGCCAGC	22	CCTGGGTCAATCCTTGGGGCCCAGACTGAGCACG[T]GATGGCAGAGGA	GGG	Sense
ExampleLocus_delT	-26	GACGCCCTCTGGAGGAAGCA	TGCTTCCTCCAGAGGGCGTC	26	TGAGCACG[T]GATGGCAGAGGAAAGGAAGCCCTGCTTCCTCCAGAGGG	GGG	Antisense
ExampleLocus_delT	-27	gCGACGCCCTCTGGAGGAAGC	GCTTCCTCCAGAGGGCGTCGC	27	GAGCACG[T]GATGGCAGAGGAAAGGAAGCCCTGCTTCCTCCAGAGGGC	AGG	Antisense
ExampleLocus_delT	-51	gCCTGGCCTGGGTCAATCCTT	AAGGATTGACCCAGGCCAGGC	23	GCCTGGGTCAATCCTTGGGGCCCAGACTGAGCACG[T]GATGGCAGAGG	GGG	Sense
ExampleLocus_delT	-52	gCCCTGGCCTGGGTCAATCCT	AGGATTGACCCAGGCCAGGGC	24	GGCCTGGGTCAATCCTTGGGGCCCAGACTGAGCACG[T]GATGGCAGAG	TGG	Sense

B

Output Table

	Variant	Protospacer	EditPos.	PAM	Base.Editor
1	Example Variant C>T edit	GGCCCCAGAGCCTCTTTCTG	6	AGG	BE3-R33A
2	Example Variant C>T edit	GGCCCCAGAGCCTCTTTCTG	6	AGG	BE3 (hA3A-R128A)
3	Example Variant C>T edit	CCCAGAGCCTCTTTCTGAGG	3	AGG	Target-AID
