# Supplementary figures and images for: PnB Designer: a web application to design prime and base editor guide RNAs for animals and plants
Source: BMC Bioinformatics. 2021 Mar 2;22:101. doi: 10.1186/s12859-021-04034-6 (PMC7923538; doi:10.1186/s12859-021-04034-6)

Sup. Fig. 2

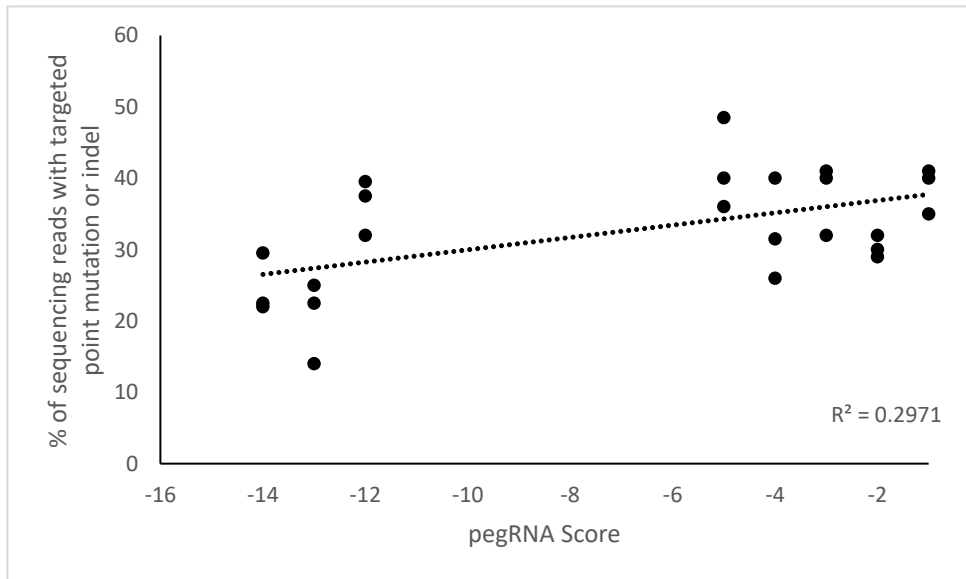

Supplement: Supplementary file 2 — Additional file 2. Fig. 2. Scatterplot, showing ‘pegRNA Score’ vs. editing efficiency of reported pegRNAs. Reported pegRNAs from Anzalone et al. Fig. 4a with their respective average editing efficiency plotted against their pegRNA Score as calculated by PnB Designer. A linear regression was fitted to investigate the relationship between these two variables and a R2 value of 0.297 was obtained. Figure was made using MS Excel (2016). [file 12859_2021_4034_MOESM2_ESM.pdf]
